# Highly Efficient and Wide Range Humidity Response of Biocompatible Egg White Thin Film

**DOI:** 10.3390/nano11071815

**Published:** 2021-07-13

**Authors:** Hafiz Mohammad Mutee Ur Rehman, Muhammad Muqeet Rehman, Muhammad Saqib, Shenawar Ali Khan, Maryam Khan, Yunsook Yang, Seongwan Kim, Sheik Abdur Rahman, Woo-Young Kim

**Affiliations:** 1 Faculty of Applied Energy System, Major of Electronic Engineering, Jeju National University, Jeju 63243, Korea; mutee1990@jejunu.ac.kr; 2 Department of Electronic Engineering, Jeju National University, Jeju 63243, Korea; saqibmuhammad@jejunu.ac.kr (M.S.); shenawaralikhan@jejunu.ac.kr (S.A.K.); yunsuk0001@jejunu.ac.kr (Y.Y.); pea8543@jejunu.ac.kr (S.K.); abdurrahman@jejunu.ac.kr (S.A.R.); 3 Department of Electrical Engineering, GIK Institute, Topi 23460, Pakistan; maryum.khan.1993@gmail.com

**Keywords:** biopolymer, humidity sensor, health monitoring, egg white, controlled measurement setup

## Abstract

Biopolymers are a solution to solve the increasing problems caused by the advances and revolution in the electronic industry owing to the use of hazardous chemicals. In this work, we have used egg white (EW) as the low-cost functional layer of a biocompatible humidity sensor and deposited it on gold (Au) interdigitated electrodes (IDEs) patterned through the state-of-the-art fabrication technology of thermal vacuum evaporation. The presence of hydrophilic proteins inside the thin film of EW makes it an attractive candidate for sensing humidity. Usually, the dependence of the percentage of relative humidity (%RH) on the reliability of measurement setup is overlooked for impedimetric humidity sensors but we have used a modified experimental setup to enhance the uniformity of the obtained results. The characteristics of our device include almost linear response with a quick response time (1.2 s) and fast recovery time (1.7 s). High sensitivity of 50 kΩ/%RH was achieved in the desirable detection range of 10–85%RH. The device size was intentionally kept small for its potential integration in a marketable chip. Results for the response of our fabricated sensor for dry and wet fingertips, along with determining the rate of breathing through the mouth, are part of this study, making it a potential device for health monitoring.

## 1. Introduction

Environmental sensors are becoming significantly important in applications related to our daily life for controlling and monitoring various ecological parameters, such as temperature, pressure, light intensity, humidity, and various gases [1,2,3,4]. Among these environmental sensors, humidity sensors are widely used in different industries, including food [5], pharmaceuticals [6], textiles [7], electronics [8], agriculture [9], etc. Humidity sensors can be classified into various types, such as resistive, capacitive, and optical fiber, with certain advantages and drawbacks associated with each type. For example, optical fiber-based humidity sensors have the advantage of not being affected by electromagnetic interference (EMI), while their drawbacks include difficult installation [10,11]. The choice of the right functional material for humidity sensors with the desirable properties of high sensitivity, stability, low cost, quick response time, wide range, and low power consumption is, therefore, currently a major research area. Humidity-sensing properties of various functional materials, such as polymers [12], inorganic semiconductors [13], and their composites [14], have already been evaluated to great effect but due to certain drawbacks of these synthesized materials, researchers have begun to explore the sensing behavior of natural materials or biomaterials. Biomaterials offer a green solution for the increasing problems caused by non-environmentally friendly materials that are synthesized in chemical laboratories [15]. Biomaterials possess several advantages over their rival functional materials, including their non-hazardous nature, renewability, biocompatibility, bioresorbablity, low cost, and self-decomposition [15]. Furthermore, these biomaterials are naturally available while other semiconductor materials must be synthesized in state-of-the-art laboratories equipped with modern technology.

Biomaterials have already been successfully tested as the functional layer for different types of electronic devices, including resistive random-access memory (RRAMs) [16,17], field effect transistors (FETs) [18], dye-synthesized solar cells (DSCs) [19], actuators [20], sensors [15], and batteries [21]. Li et al. [22] fabricated an organic inverted solar cell by using L-arginine (L-Arg) as the electron transport layer. Their device showed better performance in terms of power conversion efficiency, short current density, and open circuit voltage. Zhu et al. [23] fabricated a natural silk protein-based flexible and highly transparent electronic sensor on human skin for multi-sensing purposes. Zhai et al. [24] developed a pH-sensitive sensor (in the range of 2–12) by using gelatin as the biocompatible functional layer for application in food packaging. Toh et al. [25] used protein in a biosensor for sensing hydrogen peroxide (H2O2). Wang et al. [26] investigated the potential application of orange peel powder in an environmentally friendly non-volatile memory device with a switching ratio of 10^3^. The above reports clearly show the importance of using biopolymers as the functional layer of various electronic devices due to their immense potential of providing a green solution to the increasing toxic effects caused by the electronic industry, but not many researchers have used these natural materials for the application of sensing humidity.

Sisman et al. [27] fabricated a humidity sensor by using chitosan-functionalized tin oxide nanowires (SnO_2_ NWs) as the active layer, however, this functional layer was not completely biocompatible due to the addition of SnO_2_ NWs as the biopolymer of chitosan alone was not able to efficiently sense humidity. Natali et al. [28] studied the humidity response of a keratin-based sensor by fabricating a flexible microelectrode array but it showed low sensitivity and unsatisfactory stability. Hamouche et al. [29] also measured the humidity response of keratin by comparing the performance of two capacitive sensors with a planar structure fabricated through the dip coating method. The response of this biomaterial-based sensor was better owing to the porous and hydrophilic nature of the biopolymer. The sensor with spiral-shaped electrodes showed high sensitivity and larger capacitance. Bibi et al. [30] reported the humidity response of wheat gluten for the application of food packaging in the frequency range of 30–1000 MHz. This biopolymer showed a humidity response in the wide range of 20–95% owing to the change in its dielectric permittivity.

Among four different classes of biomaterials, including carbohydrates, nucleic acids, green plants, and proteins, egg white (EW) has shown interesting characteristics in different electronic devices, such as solar cells [31], super capacitors [32], memory devices [33], and sensors [34]. Liu et al. [32] used graphene foams templated with EW as the electrode material for a high-performance supercapacitor. Kelkar et al. [31] fabricated a highly efficient and durable quasi-solid dye-synthesized solar cell (DSSC) by using functionally engineered EW as the functional layer. Yan et al. [33] fabricated a bio-RRAM based on EW to store digital data and successfully used it for the application of mimicking the human brain. Khan et al. [34] reported an all-printed egg albumen (EA)-based humidity sensor with a wide range and high capacitance sensitivity. A few drawbacks of this work included its high impedance (4 MΩ), mainly due to the choice of Ag for patterning IDEs and using EA diluted in deionized water. Furthermore, this EA-based humidity sensor was only used for monitoring the humidity response in controlled environment and for limited applications such as health monitoring.

In this work, we have explored the humidity response of EW as the functional layer of a humidity sensor by spin coating a thin film on a transparent glass substrate on the surface of gold (Au) interdigital electrodes (IDEs) patterned through the state-of-the-art fabrication technology of thermal vacuum evaporation. The fabricated humidity sensor was used successfully for monitoring human health with promising characteristics, including high sensitivity in a wide humidity range with high repeatability. This efficient performance could be attributed to the presence of crosslinked amino acids connected with each other through peptide bonds inside the EW thin film, forming long chains. These amino acids are bonded to each other by various types of chemical bonds, including hydrogen bonds, ionic bonds, hydrophobic bonds, etc. These intra/inter-molecular chemical bonds present in the crosslinked amino acids are hydrophilic in nature and, therefore, play a vital role in sensing humidity changes in the environment.

## 2. Experimental Methods

### 2.1. Materials and Methods

**Figure 1:**
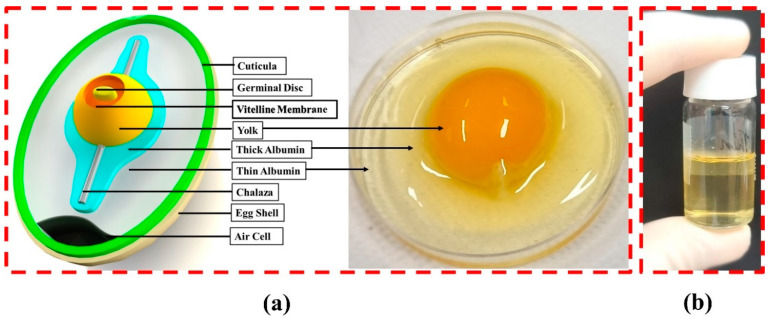
(**a**) Schematic diagram of an egg with image of a chicken egg; (**b**) image of egg white dissolved in deionized water.

First, 99.9% pure gold was used for patterning Au IDEs. EW was extracted from a fresh chicken egg that was randomly picked from the market and was used as the functional layer of the humidity sensor. EW was carefully separated from the egg yolk in a clean laboratory with the help of a stainless-steel spoon. The separated EW was collected in a test bottle and mixed with highly purified deionized water with the help of a pipette. Pure EW was not preferred because it could not form a uniform thin film owing to its high viscosity. The dilute solution of EW was bath sonicated for 10 min and dispersed thoroughly, followed by filtration to remove any solid particles. It was found that the raw EW was highly soluble in deionized water owing to the presence of vitamins, fats, salts (Fe^+^, K^+^ and Na^+^), and nearly forty proteins (ovalbumin, conalbumin, ovomucoide, lysozyme, etc.). The schematic diagram of a chicken egg with an image of a chicken egg is shown in Figure 1a, while Figure 1b shows an image of a test bottle containing EW dissolved in deionized water.

### 2.2. Sensor Fabrication

**Figure 2:**
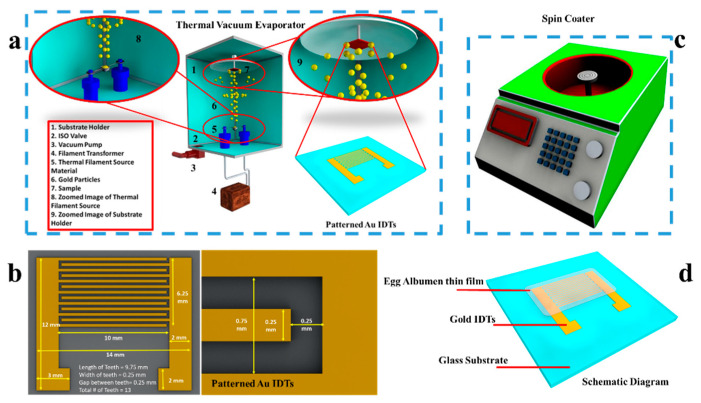
Fabrication of egg white-based humidity sensor. (**a**) Schematic diagram of thermal vacuum evaporation. (**b**) Images of fabricated Au IDTs patterned on transparent glass substrate with labeled dimensions. (**c**) Spin coater for depositing thin film of egg white. (**d**) Schematic diagram of biomaterial-based humidity sensor.

The glass substrate was cleaned sequentially with ethanol, acetone, and deionized water, to remove dust particles and contaminants from its surface. The cleaned substrate was treated with UV ozone for 10 min to enhance the adhesion of its surface and make it more hydrophilic. We opted for patterning the planar structure of IDEs over other reported electrode patterns for sensing humidity as they were expected to offer higher sensitivity to the change in characteristics of the biopolymer-based functional layer with respect to change in relative humidity (RH%). IDEs can be considered as two chemristors connected in a parallel combination. The state-of-the-art fabrication technology of thermal vacuum evaporation was used for patterning Au IDEs owing to its high controllability and extremely efficient results, and a schematic diagram is shown in Figure 2b. A thin layer (10 nm) of chromium was deposited on the glass substrate for better adhesion of Au IDEs on its surface. The thickness of Au IDEs was 50 nm. Metallic electrodes were patterned at a pressure of 2 × 10^−6^ Torr while the deposition rates of the Cr layer and Au IDEs were kept constant at 1 A/sec and 2 A/sec, respectively. The spacing and width of each finger of an IDE directly affect the performance of a humidity sensor, therefore, we optimized these dimensions, as shown in Figure 2b with detailed labeling of each part. The patterned Au IDEs are shown in the figure. These dimensions of the IDEs were the smallest that could be achieved by using our proposed fabrication technology. The smallest dimensions were preferred to highlight the potential of our device for integration in CMOS technology. The diluted ink solution of EW in deionized water was spin coated at 3500 rpm for 40 s to complete the device fabrication process of the biopolymer-based humidity sensor, as shown in Figure 2c. A complete schematic diagram of our fabricated humidity sensor is shown in Figure 2d.

### 2.3. Sensor Characterization

The biomaterial-based sensor and its thin film were characterized to find their structural, morphological, and electrical characteristics. These characterizations were helpful in determining the conduction mechanism effectively. The surface roughness of the functional layer was measured through a 3D nanoprofiler (Nano View 2000 universal non-contact surface profiler) in phase shifting interferometry (PSI) phase. Dimensions of patterned Au IDEs were determined by using an optical microscope (Olympus BX60, Tokyo, Japan). The contact angle of the spin coated thin films was measured using a contact angle tester (Drop Shape Analysis DSA30, Kruss, Germany). The chemical composition and structure of the EW biopolymer were determined by using Fourier transform infrared (FTIR) spectroscopy (ALPHA II FTIR spectrometer).

**Figure 3:**
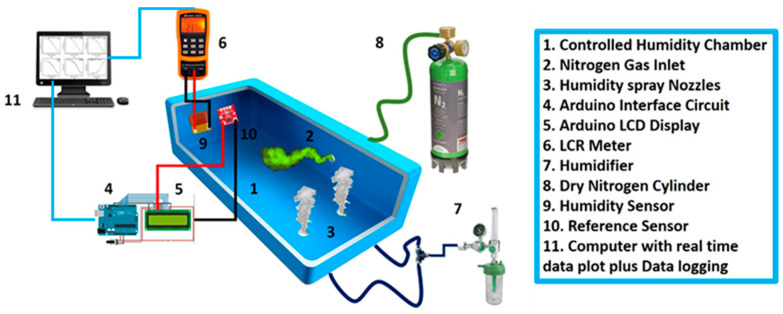
Schematic diagram of highly controllable measurement setup for obtaining the impedance data of the developed humidity sensor with thorough labeling of each part.

The change in values of the impedance response relative to the change in humidity were recorded with the help of a custom designed and highly controllable sealed environmental chamber, whose schematic diagram is shown in Figure 3 with complete labeling of each part. A reference sensor and humidity sensor were installed on the same side next to each other inside the sealed chamber. Inlet for the inflow of N_2_ gas to dehumidify the sealed chamber was located on the perpendicular wall of the chamber at a 15 cm distance from both the sensors, while the inlet for the humidifier was located at the bottom of the chamber at 17.5 cm from both sensors. The reason for maintaining this distance between the inlets and sensors for humidification and dehumidification was to avoid sudden changes in the level of humidity inside the sealed chamber. Real values of relative humidity (RH%) were recorded with the help of a reference sensor (HTU-21D) that was placed near the EW-based humidity sensor to maintain the desired level of humidity inside the measurement chamber through a feedback signal. An LCR meter (Applent AT-825) was used at a 1 kHz frequency with an AC output of 0.6 V_rms_ to measure the impedance values. The measurement chamber was completely sealed, and its humidity level was decreased to 0 RH% from ambient conditions with the help of a controlled inflow of N_2_ purging gas through the nozzle. Atomized water vapor was used to gradually increase the humidity level inside the sealed chamber with the help of a humidifier, while its temperature was maintained at 25 °C to avoid the effect of temperature on humidity measurements.

## 3. Results and Discussion

### 3.1. Morphological and Chemical Characterizations

**Figure 4:**
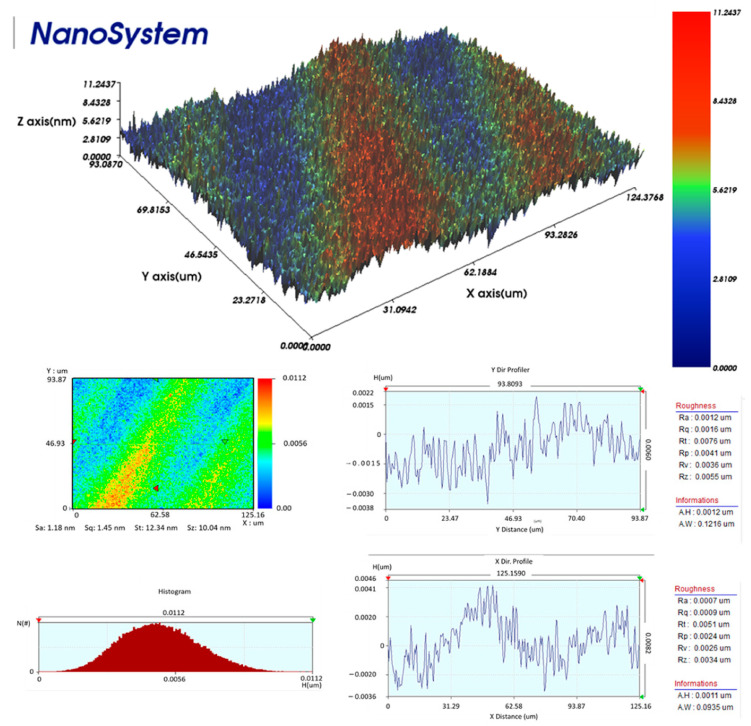
Morphological characterization results obtained from 3D nanoprofiler illustrating surface roughness of egg white thin film.

**Figure 5:**
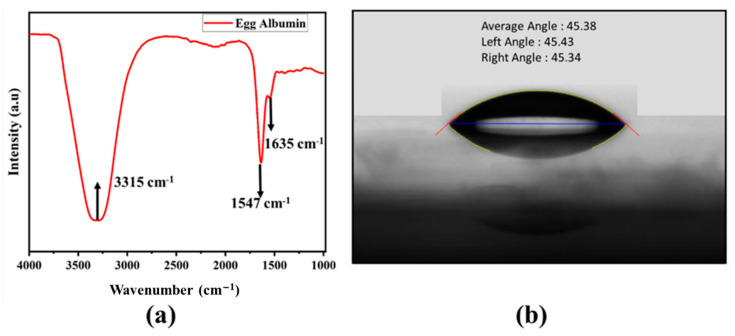
(**a**) Chemical composition and structure of functional layer depicted in FTIR spectrum of EW. (**b**) Contact angle of EW thin film showing its hydrophilic nature.

Figure 4 shows the 3D nanoprofile image of the EW thin film, illustrating its highly uniform surface with average roughness of 12 nm. The chemical structural analysis of egg albumin was carried out using an FTIR spectrophotometer, as shown in Figure 5a. The absorption band at 3315 cm^−1^ corresponds to O–H stretching vibrations. The absorption peak at 1635 cm^−1^ corresponds to the amide-I (N–H) region of egg albumin due to C=O stretching vibrations; there is another peak at 1547 cm^−1^ in the FTIR spectra which corresponds to the amide-II region due to N-H bending in plane and C–N stretching. These characteristic peaks are in good agreement with already reported IR spectra of egg albumin [35]. Furthermore, the contact angle of the EW thin film was determined to assess its hydrophilic property, as shown in Figure 5b. This is an important test to determine the wettability of the functional material. The measured static contact angle of EW was 45°, indicating that this biomaterial can easily absorb water molecules.

### 3.2. Electrical Characterizations

The electrical response of the EW thin film-based humidity sensor was recorded by keeping it close to the reference sensor inside the sealed measurement chamber. Electrical contacts were made by using silver (Ag) epoxy to make firm contact with the Au IDEs. The RH% was decreased to 0% with the help of N_2_ gas and increased gradually from 0–100% RH with the help of a humidifier and the resulting values of impedance were recorded. The thin film of EW-based humidity sensor exhibited an inversely proportional relation between its impedance and RH, as shown in the electrical characteristic graph, which is consistent for each set of recorded data.

The results show that the sensor does not respond to changes in RH% from 0–10% RH and its impedance begins to decrease gradually after 10% RH. The sensor showed an almost linear decrease in the impedance values, which is highly desirable for humidity sensors. The sensor did not respond to changes in the water content of its environment at RH > 85%, which signifies that the EW sensor has a wide sensing range of 10–85% RH. The drop in impedance values was mainly due to a decrease in the resistance of the EW thin film which may have been due to the rapid adsorption of water molecules in the porous surface of the biopolymer.

**Figure 6:**
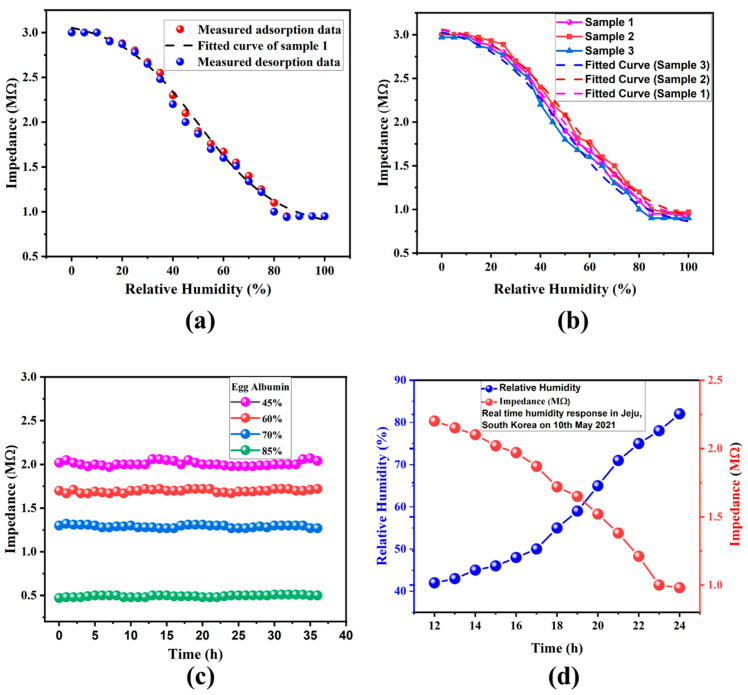
(**a**) Impedance response of egg white-based humidity sensor with change in RH%. (**b**) Impedance response of multiple egg white-based sensors, showing their reliability. (**c**) Stability of egg white-based humidity sensor at four different levels of RH%. (**d**) Real-time humidity response of fabricated sensor for 13 h in the open environment of Jeju on a rainy day.

EW is considered a good insulator and, therefore, it has high impedance values in dry conditions. Experiments have shown that the impedance value is highly dependent on the water content absorbed by this material, which makes it a useful functional material for sensing humidity. Adsorption of water molecules in the thin film of EW may dissolve organic material in the cells, resulting in the easy flow of the current and reduction in the impedance value. Water itself is a good conductor of electric currents, therefore, its successful penetration into a thin film of an insulating biopolymer will have a definite effect on its impedance values. A thin film of EW tries to attain equilibrium when there is a difference in the content of water vapor in its surrounding environment by allowing the water molecules to diffuse through it. The obtained results show that the impedance value decreases almost linearly with increasing RH%, as shown in Figure 6a. A total of three identical samples were fabricated by the same technology that were characterized in similar conditions. The impedance for each sensor varied from 3 MΩ to 0.3 MΩ for the RH% range of 10% to 85%. The results suggest that 85% RH is the saturation point of our sensor because no change in the impedance value was recorded after this point, signifying it to be the maximum detection limit. The recorded impedance values clearly showed overlapping behavior, demonstrating that highly repeatable and reliable nature of these high-quality results, as shown in Figure 6b. The physiosorbed layer of water acts as a liquid at higher levels of humidity (>85%), which results in a saturation point. The measured values of the humidity sensor were linearly fitted by using the equation of Boltzmann curve fitting (R^2^ > 0.99) that allows easy conversion of the sensor’s response to relative humidity by solving the equation with high accuracy. The stability of the biopolymer-based humidity sensor was also recorded at four different RH% levels (45%, 60%, 70%, 80%) and the obtained results exhibited highly stable output for more than 36 h, as shown in Figure 6c. The real-time response of our sensor was recorded by keeping it in an open air environment in Jeju, South Korea for thirteen hours on a rainy day on which humidity varied from 45% to 82%. The data were recorded every hour from 12:00 p.m. to 1:00 a.m. The obtained results showed that the EW sensor was highly efficient, as shown in Figure 6d, as its values varied quickly with a quick response time over a wide humidity range.

Chemical characteristics of amino acids, such as hydrophobic, hydrophilic, basic, and acidic, are determined based on the side chains. Linkage between COOH and NH_2_ forms a peptide bond among multiple amino acids, resulting in water release, and for this reason it is known as a dehydration reaction. Amino acids exist in four different forms depending on the formation and bonds between their long chains. Peptide and disulfide bonds are the strong bonds which protect EW from the process of denaturation, thus providing high thermodynamic stability. The denaturation of proteins modifies the paths for oxygen to diffuse, hence resulting in a reduction in oxygen scattering and an increase in the probability of forming conductive channels throughout the EW thin film. The porous structure of EW provides a large surface area for the dispersion and transmission of water molecules to become adsorbed. To understand the conduction mechanism of charge carriers taking place inside the functional layer of the proposed humidity sensor, it is important to understand the chemical structure of EW. It is composed of proteins that contain long chains of amino acids. These proteins are recognized based on these amino acid chains, consisting of a carboxylic acid group and central alpha (α) carbon part that is connected to one of the side chains and hydrogen (H).

Physical adsorption of water vapor in the thin film of EW results in decreasing its impedance because the oxygenated hydrophilic groups are ionized due to the presence of a highly electrostatic field. This ionization process results in the formation of hydronium ions (H_3_O^+^) in large quantities. Proton hopping takes place between two adjacent molecules of water, as depicted by the equation of the Grotthuss reaction. Thus, the hydrophilic nature of the EW thin film and its humidity-sensing behavior can be linked to the side chains attached to these disulfide and peptide bonds. As a result of these chemical reactions, thio and amino acids lose H atoms, resulting in covalent bonding between two S atoms of distinct amino acids. Loss of a couple of H atoms results in a decrease in the concentration of charge carriers because each H atom consists of one electron and hole pair. There are various amino acids present in EW but only six of them are the cause of its hydrophilic nature, owing to their polar side chains. The reaction of humidity with these polar chains causes a change in the capacitance and impedance values of EW that can be used for sensing humidity changes. Threonine and serine are two of the polar amino acids whose hydrophilic property is due to the alcohol functional group as these groups can form hydrogen bonds with the water molecules present in the environment of the sensor. Cysteine plays a critical role to form quaternary and tertiary protein structures and it exhibits a hydrophilic nature due to its parallel chemical bonds between a hydroxyl group and sulfhydryl group. Side chain reactions of cysteine enable it to form a bond between two S atoms through oxidation. Furthermore, due to the neutral nature of NH_2_ bonded with a carbonyl group in standard conditions, the formation of an amide takes place, causing its hydrophilic behavior. Among the amino acids, proline has a somewhat different nature as it is a part of polypeptide chain and has no H atom attached to its N atoms, thus it does not have any polarity.

Adsorption of water molecules into the thin film of a biopolymer is a two-step process. In the first step of water adsorption, water molecules form hydrogen bonds with the hydroxyl (OH) groups present in EW, and due to this, no protons can take part in the conduction process, owing to the restriction caused by hydrogen bonds. Water molecules continue to be adsorbed on the surface of the EW thin film to form a less ordered second layer of water molecules on its surface due to the presence of local hydrogen bonds. The porous structure of an EW thin film biopolymer might cause a large contact area between moisture and hygroscopic groups present on the surface of the EW, resulting in faster transmission and diffusion of water molecules in the functional layer of the humidity sensor. With the passage of time, more layers of water molecules keep forming without any order, known as the physisorption process, thus making room for the protons to take part in the conduction process, as explained by the Grotthuss mechanism [36]. Apart from protons, more ions of H_3_O^+^ and OH^−^ are also formed, which increases the ionic conductivity, resulting in a decrease of impedance values with an increase in relative humidity, as shown by the following set of chemical reactions.
(H_2_O → H^+^ + OH^−^, H_2_O + H + → H_3_O^+^, H_2_O + H_2_O 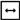 H_3_O^+^ + OH^−^)

Conduction at a relatively low level of RH% is primarily based on H^+^ jumping owing to the low absorption rate of water molecules. Increasing the RH% level in the surroundings of the humidity sensor results in the creation of more H^+^ ions and the formation of hydrogen bonds between the biopolymer and water molecules, which accelerate their flow in the functional layer.

### 3.3. Transient Response and Applications of the Sensor

**Figure 7:**
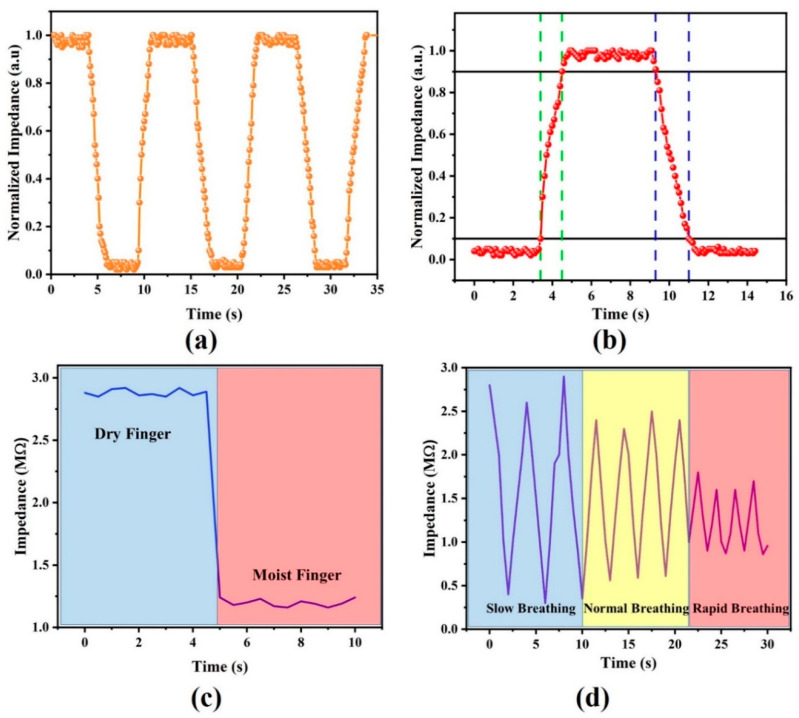
(**a**) Multiple cycles of transient response showing high repeatability of egg white-based humidity sensor. (**b**) Response and recovery time of egg white-based humidity sensor as depicted from a single cycle of transient response. (**c**) Response of egg white-based humidity sensor for dry and moist finger. (**d**) Application of egg white-based humidity sensor for monitoring health, showing its ability to distinguish between slow, normal, and rapid breathing rates.

The response time is 10% to 90% of the total change in impedance value, while recovery time can be defined as the time required to drop from 90% of the maximum impedance value to 10% of the maximum impedance value. The transient response of our sensor was recorded to evaluate its performance for different applications, as shown in Figure 7a. Repeatability of its multiple cycles indicated high dependability and accuracy. A single cycle of the transient response was further used to evaluate two of the most important parameters of any humidity sensor, i.e., response time and recovery time, as shown in Figure 7b. Response time and recovery time of our sensor were recorded within the measurable range by selecting two different levels of RH%. The response time (1.1 s) of our sensor was less than its recovery time (1.7 s), indicating a faster adsorption rate than desorption rate of water molecules. This response time is much faster than the previously reported response times of other humidity sensors in the literature [10,37]. This fast response and recovery time could be due to the use of highly conductive Au IDTs and the thin film of the functional layer that have a lower capability to trap water molecules. The digital data of impedance values were logged and recorded with a resolution of 0.1 s on a computer. The average sensitivity of the EW-based humidity sensor was 50 kΩ/RH%. Although there is not much difference in the response time and recovery time of the EW-based humidity sensor, this slight difference can be attributed to the highly hydrophilic nature of EW and the surface roughness of the deposited thin film that allows water molecules to quickly adsorb on its surface. The impedance value changes by 10 orders (3 MΩ to 0.3 MΩ) of its original value, which provides a large range for sensing humidity. The reason for relatively larger drop in impedance (10 orders) can be attributed to the extremely hydrophilic nature of EW that provides enhanced affinity towards even low concentrations of water molecules.

The fast response and recovery time of our sensor enables it to detect the rate of breathing and categorize it as normal (average person), fast (athlete), and slow (patient), so that one can check the status of respiration. The performance of our fabricated sensor was also determined for the application of monitoring human health. Real-time monitoring of the human respiratory system can play a vital role in providing intensive health care to patients. The surface of the developed sensor was exposed to human breathing to check its sensitivity. The obtained results were encouraging, with a quick response and recovery time and the ability to sense even small changes in RH%. Conductivity of the functional layer was increased with exhaled human breath due to the presence of water content. Resistance of the functional layer increased while inhaling due to a decrease in water content, hence proving the application of the EW thin film for monitoring human health. The literature suggests that exhaling air at a slow speed has a larger amount of moisture content as compared to breathing at a rapid speed. This difference in the level of humidity can be sensed by our sensitive humidity sensor by monitoring the variance in the value of its impedance, as shown in Figure 7c. The Figure 7d shows the response time of respiration for a 30-year-old person with a slow (2.3 s), normal (1.6 s), and rapid (1 s) breathing rate, respectively.

We tested our device for the application of distinguishing between a moist and dry finger by applying hand cream on a fingertip and moving it close to our sensor. The change in the impedance value for the wet and dry finger was due to the absorption of water molecules by the EW thin film generated from the surface of fingertip and these water molecules were desorbed upon lifting the fingertip, as shown in Figure 7d. The high sensitivity of our sensor made it possible to recognize the difference between a moist and dry finger. It was also observed that impedance values dropped with the decrease in distance between the fingertip and the surface of the sensor. Successful implementation of this experiment with our biopolymer humidity sensor makes it an attractive device for human–machine interface (HMI) applications. The obtained results of our reported biomaterial-based EW humidity sensor are highly encouraging to commercialize it as a self-decomposing humidity sensor by using simple industrial fabrication technologies at a low cost to provide a green solution to the increasing toxic problems due to the use of hazardous chemicals in the electronic industry.

## 4. Conclusions

In summary, a biocompatible thin film of egg white (EW) biopolymer was prepared by diluting EW with deionized water and depositing it on Au IDTs to fabricate a humidity sensor. Various characterizations of EW, including surface roughness (3D nanoprofiler), FTIR, contact angle, and electrical response, were investigated in detail to understand its humidity-sensing behavior. The sensor showed an almost linear response, short response/recovery time (response time of 1.1 s, recovery time of 1.7 s), high average sensitivity (50 KΩ/%RH), and a wide range relative humidity response (10–85%RH) by 10 orders of magnitude (3–0.3 MΩ). Due to the wide range of our humidity sensor, it can indicate uncomfortable humidity levels of <30%RH and >75%RH after attaching an additional circuit to it. We tested our fabricated EW-based humidity sensor for different applications, including environmental monitoring, breath monitoring, and skin care. The obtained results were highly encouraging for various aspects of health monitoring with fast response time and high sensitivity.

## Data Availability

The data presented in this study are available on request from the corresponding author.
